# HEart and BRain interfaces in Acute ischemic Stroke (HEBRAS) – rationale and design of a prospective oberservational cohort study

**DOI:** 10.1186/s12883-015-0458-2

**Published:** 2015-10-22

**Authors:** Karl Georg Haeusler, Ulrike Grittner, Jochen B. Fiebach, Matthias Endres, Thomas Krause, Christian H. Nolte

**Affiliations:** Center for Stroke Research Berlin, Charité - Universitätsmedizin Berlin, Berlin, Germany; Department of Neurology, Campus Benjamin Franklin, Charité - Universitätsmedizin Berlin, Hindenburgdamm 30, 12200 Berlin, Germany; Excellence Cluster NeuroCure, Charité - Universitätsmedizin Berlin, Berlin, Germany; German Center for Neurodegenerative Diseases, Berlin, Germany; German Center for Cardiovascular Diseases, Partner Site Berlin, Berlin, Germany; Berlin Institute of Health, Berlin, Germany; Department for Biostatistics and Clinical Epidemiology, Charité – Universitätsmedizin Berlin, Berlin, Germany

**Keywords:** Atrial fibrillation, Cardiac MRI, ECG monitoring, Ischemic stroke, Heart rate variability, Autonomic dysfunction

## Abstract

**Background:**

An effective diagnostic work-up in hospitalized patients with acute ischemic stroke is vital to optimize secondary stroke prevention. The *HEart and BRain interfaces in Acute ischemic Stroke* (HEBRAS) study aims to assess whether an enhanced MRI set-up and a prolonged Holter-ECG monitoring yields a higher rate of pathologic findings as compared to diagnostic procedures recommended by guidelines (including stroke unit monitoring for at least 24 h, echocardiography and ultrasound of brain-supplying arteries).

**Methods/Design:**

Prospective observational single-center study in 475 patients with acute ischemic stroke and without known atrial fibrillation. Patients will receive routine diagnostic care in hospital as wells as brain MRI, cardiac MRI, MR angiography of the brain-supplying arteries and Holter-monitoring for up to 10 days. Study patients will be followed up for cardiovascular outcomes at 3 and 12 months after enrolment.

**Discussion:**

By comparing the results of routine diagnostic care to the study-specific MRI/ECG approach, the primary outcome of HEBRAS is the proportion of stroke patients with pathologic diagnostic findings. Predefined secondary outcomes are the association of stroke localization, autonomic dysbalance and cardiac dysfunction as well as the effect of impaired heart-rate-variability on long-term clinical outcome.

The investigator-initiated HEBRAS study will assess whether an enhanced MRI approach and a prolonged ECG monitoring yield a higher rate of pathological findings than current standard diagnostic care to determine stroke etiology. These findings might influence current diagnostic recommendations after acute ischemic stroke. Moreover, HEBRAS will determine the extent and clinical impact of stroke-induced cardiac damage.

**Trial registration:**

Clinicaltrials.gov NCT02142413.

## Background

Variations and delays in the diagnostic procedures during hospitalization after acute ischemic stroke are common [1–3]. At the same time, stroke etiology remains cryptogenic in about 20–25 % of stroke unit patients [4].

Recent studies have shown that magnetic resonance imaging (MRI) and MR angiography are able to detect cardiac [5, 6], aortic [7] as well as carotid sources of embolism with comparable sensitivity as compared to diagnostic ultrasound [8]. Moreover, prolonged ECG monitoring can significantly increase the detection rate of atrial fibrillation (AF) [9, 10]. The detection of AF implies a four- to fivefold increased risk of stroke [11]. These developments may allow for a more effective diagnostic work-up in patients with acute ischemic stroke as compared to standard diagnostic procedures, namely ultrasound of the brain-supplying arteries, echocardiography and stroke unit monitoring. However, there is no common standard of stroke unit monitoring [12] and guidelines [13] do not strictly recommend prolonged ECG monitoring [14]. In addition, availability of echocardiography is often limited [1, 15], and a substantial number of stroke patients are not willing or able to undergo semi-invasive transesophageal echocardiography, while transthoracic echocardiography is not sufficient for assessing aortic plaque and left atrial thrombi.

The prospective observational *HEart and BRain interfaces in Acute ischemic Stroke* (HEBRAS) study therefore aims to assess the detection rate of pathologic findings relevant to stroke etiology as obtained by an enhanced MRI set-up (including brain MRI, cardiac MRI, MR angiography of the brain-supplying arteries) and a prolonged, non-invasive, commonly available Holter monitoring (of up to 10 days duration) in comparison to findings obtained by routine diagnostic procedures after acute stroke. In addition, the underlying pathophysiological mechanisms and the prognostic impact of stroke-induced cardiac damage are still poorly understood [16, 17]. For that reason, the investigator-initiated HEBRAS study attempts to shed more light on the relationship between stroke localization (e.g., insular involvement), observed cardiac damage (as indicated by troponin elevation) and the activation of the autonomic nervous system (as indicated by impairment of heart rate variability and elevated urinary norepinephrine levels). Moreover, stroke-induced autonomic dysfunction and observed cardiac damage will be analyzed with respect to functional outcome, mortality, recurrent stroke and myocardial infarction during a one year follow-up.

## Methods

### Study design

The Center for Stroke Research Berlin (CSB) is the sponsor of the investigator initiated HEBRAS study, which is supported by a grant from the German Federal Ministry of Education. The study was approved by the scientific advisory board of the CSB in September 2013, and by the Ethics Committee of the Charité - Universitätsmedizin Berlin, Germany (EA2/033/14) in March 2014. All study procedures are carried out in accordance with the principles of Good Clinical Practice and the Declaration of Helsinki. All study patients have to fulfill the study entry criteria listed in Table 1 including written informed consent.

**Table 1:** Inclusion and exclusion criteria of the HEBRAS study

Inclusion criteria
• Written informed consent by patient
• Age ≥ 18 years.
• Acute ischemic stroke (with matching brain lesion on MR imaging).
• Admission to the stroke unit at the Charité, Campus Benjamin Franklin
• Enrolment within 144 h after onset of stroke-related symptoms.
Exclusion criteria
• Known AF by past medical history before hospital admission.
• AF according to 12-lead ECG on hospital admission.
• AF according to inpatient ECG recording / stroke unit monitoring before enrolment.
• Pre-stroke life expectancy less than 1 year.
• Participation in an interventional study.
• Pregnancy and / or breast-feeding.
• Contraindications to undergo MRI (i.e., mechanic heart valve, cardiac pacemaker)
• History of adverse response to MRI contrast agents
• Clinically severe heart failure (NYHA III-IV) [37]
• Renal insufficiency (creatinine > 1.3 mg/dl (females); creatinine > 1,7 mg/dl (males))

Study patients will receive routine diagnostic procedures in hospital (defined as stroke unit monitoring (according to clinical needs), echocardiography, ultrasound of the brain-supplying arteries, and 24-hour Holter-ECG (if indicated according to the treating physician)). Moreover, study patients will undergo an enhanced MRI work-up (comprising brain as well as cardiac MRI, MR angiography of the aortic arc and the supra-aortic, brain-supplying arteries) combined with Holter-ECG of up to 10 days (including a maximum of 5 days after hospital discharge). For the primary outcome, the results of routine diagnostic care will be compared to study-related diagnostics. Results of the ECG core lab analysis will be provided to study patients and treating physicians. Study patients will be followed up for cardiovascular outcomes e.g., recurrent stroke, major vascular events, death and degree of dependency at 3 and 12 months after enrollment.

### Stroke etiology

Etiology will be determined according to TOAST [18] and ESUS criteria [4]. The following diagnostic findings will be regarded as pathologic and relevant to stroke etiology: atrial fibrillation of any type, sick sinus syndrome, bacterial or nonbacterial thrombotic endocarditis, ipsilateral carotid or intracranial stenosis of ≥ 50 % according to NASCET [19], left atrial or left ventricular thrombus, atrial myxoma, acute myocardial infarction resulting in wall motion abnormalities, akinetic left ventricular segment, dilated cardiomyopathy, systolic dysfunction with reduced left ventricular ejection fraction (<45 %), aortic plaque of ≥ 4 mm.

### Magnetic resonance imaging

In HEBRAS, enhanced diagnostic MRI work-up is defined as brain MRI, cardiac MRI and MR-angiography of the aortic arc and the brain-supplying arteries. All MR examinations are performed on a 3 Tesla MR scanner (TIM TRIO, Siemens, Erlangen, Germany). Cerebral MRI mainly consists of a T2*-sensitive sequence (TR 620 ms, TE 20 ms, flip angle 20°, FOV 220 mm, slice thickness 5 mm) to exclude intracerebral hemorrhage, a diffusion weighted (DWI) sequence (TR 7600 ms, TE 93 ms, FOV 230 mm, slice thickness 2.5 mm) for detection of ischemic tissue, and a 3D time-of-flight (TOF) angiographic sequence (TR 22 ms, TE 3.86 ms, flip angle 18°, FOV 200 mm, slice thickness 0.65 mm) for imaging of intracranial vessels. For cardiac imaging, initially a dark-blood–prepared HASTE (half-Fourier acquisition single-shot turbo spin-echo) sequence (TR 750 ms, TE 49 ms, Flip angle 160°, Slice thickness 5 mm) is acquired, followed by 2 chamber as well as 4 chamber view acquisitions using a cine SSFP sequence (TR 40.56 ms, TE 1.48 ms, flip angle 50°, slice thickness 5 mm) with prior scouting for individual frequency adjustment. Post-contrast imaging is performed after intravenous injection of 0.1 mmol/kg body weight Gadobutrol (Gadovist®, Bayer Schering Pharma, Berlin, Germany) with post-contrast cine imaging parameters held constant. For angiographic imaging of the aortic arch as well as extracranial cerebral arteries, a three-dimensional TWIST (Time-resolved angiography With Interleaved Stochastic Trajectories) sequence was used (TR 2.36 ms, TE 0.99 ms, Flip angle 20°, Slice thickness 1.40 mm). Finally, myocardial late enhancement is characterised employing a turbo FLASH (Fast Low Angle SHot) sequence (TR 750.00 ms, TE 1.97 ms, Flip angle 20°, Slice thickness 8 mm).

The following stratification of lesion localization is planned: left / right anterior cerebral artery, left / right middle cerebral artery, left / right posterior cerebral artery, left / right anterior choroideal artery, left / right insular cortex, left / right basal ganglia, left / right thalamus, left / right cerebellum, left / right brainstem, left / right medulla oblongata.

To bypass restrictive *a priori* assumptions on a specific relationship between stroke lesion and laboratory changes, an additional voxel-by-voxel analysis of lesion location and troponin-elevation will be conducted employing statistical principles developed for functional brain imaging [20, 21].

### Study ECG & core lab analysis

Additional ECG recording will be performed by using the portable CardioMem®4000 (GETEMED AG, Teltow, Germany). Study ECG will be started as soon as possible after enrolment and will be continued for a maximum of 10 days, i.e., two consecutive ECG measurements of five days. When patients are discharged before the first five days are completed, ECG recorder will be changed at discharge, resulting in an overall duration of less than ten days in Holter-ECG.

Study ECG data will be transmitted to the cardiac core lab at Department of Cardiology and Pneumology, Georg-August-University Göttingen, Germany for analysis. Raters analysing ECG-data will be blinded for clinical data including infarct localization. Core lab analysis will be provided to the respective study patient and the patient’s treating physician (if indicated by clinical assumption).

The following definitions will be used throughout the study: Atrial fibrillation: Absolute arrhythmia lasting ≥ 30 s as defined in the current ESC guideline [22]. Short atrial tachycardia: Atrial tachycardia lasting < 30 s and consisting of ≥ 6 conducted premature activations. Excessive supraventricular ectopic activity: More than three premature atrial complexes per hour [23]. Atrial flutter will be reported separately, and the study specifies to initiate anticoagulation for atrial flutter in the same way as for AF. The analysis will include: heart rhythm; PQ intervall [s], minimal [bpm], maximal [bpm] and mean heart rate (HR) [bpm], and measures of Heart rate variability (HRV) such as the standard deviation of beat-to-beat (NN) intervals (SD-NN), Root Mean Square of Successive Differences (rMSSD), HRV-Triangular Index (HRV-TI), low and high frequency (LF and HF, respectively) band and LF/HF Index [24].

### Baseline visit in hospital

Baseline assessment of all study patients will include: a detailed analysis of patient demographics (also including time of stroke onset, hospital admission as well as admittance to the ward), clinical characteristics (including the National Institutes of Health Scale Score [25] and the modified Rankin Scale score [26], vital signs, medical history and concomitant diseases, extend and results of inpatient ECG recording, laboratory results (all data of clinical routine assessment plus high-sensitivity (hs) troponin and NT-proBNP), results of imaging (head CT/MRI, carotid ultrasound of brain-supplying arteries, echocardiography) and TOAST classification at hospital discharge (before evaluation of the additional ECG monitoring), in hospital treatment (i.e., thrombolysis, ventilation etc.) as well as in hospital complications (such as pneumonia, recurrent stroke or myocardial infarction).

### Study follow-up

Follow-up will be done 3 months and one year after the index stroke by a telephone interview conducted by trained personal of the Center for Stroke Research Berlin, Germany. The following data will be assessed: medication, recurrent ischemic stroke and other major vascular events, major bleeds, and the mRS score. Additionally, (all-cause) death and date of death will be registered using information from registration offices.

### Study outcomes

The primary outcome of the HEBRAS study is the rate of pathologic findings relevant to stroke etiology in patients with acute ischemic stroke obtained by enhanced diagnostic MRI work-up combined with prolonged Holter-monitoring in comparison to findings obtained by the routine diagnostic work-up in this cohort of patients. The primary hypothesis is that the detection rate of pathologic findings can be increased by undergoing enhanced diagnostic MRI work-up combined with prolonged Holter-monitoring. The primary outcome and all secondary outcomes are listed in Table 2, and a flowchart of study specific data acquisition is provided in Table 3.

**Table 2:** Primary outcome and secondary outcomes of the HEBRAS study

Primary outcome
• Detection rate of pathologic findings relevant to stroke etiology in patients with acute ischemic stroke obtained by enhanced diagnostic MRI work-up combined with prolonged Holter-monitoring in comparison to findings obtained by routine diagnostic work-up.
Secondary outcomes
• To assess the benefit of prolonged continuous ECG-monitoring in AIS patients to detect paroxysmal AF.
• To determine the proportion of patients with first detected paroxysmal AF by prolonged Holter-monitoring (for up to 5 days) after hospital discharge.
• To identify the impact of stroke localization on autonomic changes (as indicated by elevated urinary norepinephrine levels and measures of HRV) or cardiac dysfunction (as indicated by troponin T serum levels).
• To identify the impact of impaired HRV on recurrent vascular events and clinical outcome after AIS at 3 or 12 months after the index stroke, respectively.
• To assess the predictive value of imaging and biomarkers for AF-detection in patients with acute ischemic stroke.

**Table 3:** Study flowchart of the HEBRAS study

	In-hospital stay				Follow-up	
	Admission	Day 1	Day ≥2	Discharge	3 months	12 months
Baseline data*	X					
Past medical history	X				X	X
Heart rate at rest (routine)	X	X	Daily	X		
Heart rhythm (routine)	X	X	Daily	X		
12-lead ECG	X					
24-hour Holter			(X)			
Additional ECG monitoring^a^	ongoing			ongoing^c^		
Respiratory rate	X	X	Daily			
Clinical signs of infection	X		X	X		
Leukocytes [per mm^3^]	X					
Creatinine [mg/dl]	X					
Potassium [mmol/l]	X					
Serum hs troponin I [ng/l]	X	X				
NT-proBNP [ng/l]		X				
Urine norepinephrine [nmol/l]		X				
Cardiac MRI		X				
MR angiography^b^		X				
Brain MRI		X				
NIHSS	X			X		
Modified Rankin scale score	X			X	X	X
Recurrent stroke, myocardial infarction or death of any cause			X	X	X	

^a^see Methods section for more details

^b^including the aortic arc and brain supplying arteries

^c^extended for up to 5 days after hospital discharge/ transfer to a rehabilitation clinic

### Statistical analysis

The sample size calculation is based on the aforementioned primary hypothesis (Table 2), that an enhanced diagnostic work-up consisting of cerebral and cardiac MRI (including contrast-enhanced angiography of the aortic arch and the carotid arteries) combined with a prolonged ECG-Monitoring (of up to 10 days duration) leads to a reduction in the number of strokes classified as cryptogenic from 30 to 25 % when compared to the routine diagnostic work-up at the Stroke Unit. A sample size of 396 patients which receive both the enhanced as well as the routine diagnostic work-up would provide a power of 79 % to detect a difference in the number of strokes classified as cryptogenic (from 30 % after routine diagnostic work-up to 25 % after enhanced diagnostic work-up, and with assumed 13 % discordant pairs: 9 % diagnosed as cryptogenic stroke by routine work-up but not according to enhanced diagnostic work-up, and 4 % diagnosed as cryptogenic stroke by enhanced work-up but not according to routine diagnostic work-up). The hypothesis will be tested using the McNemar-Test (two-sided, α = 0.05). An interim analysis (with a significance level of α1 = 0.004) will be performed when 264 patients (67 %) are enrolled in the study. If p is ≤ α1 at the interim analysis, the study will be terminated. When the study will be continued, the primary endpoint will be tested with α2 = 0.046 (two- tailed); the overall significance level will be 5 %.  Taking into account a drop-out rate of about 20 %, 475 patients will have to be enrolled in the study. 

### Study schedule

The first patient was enrolled in May 2014. We anticipate a recruitment period of 18 months, and a recruitment period of additional 12 months is intended to enroll 475 patients With regard to the planned follow-up duration, first patient in to last-patient out will be 4.5 years in total.

## Discussion

The primary aim of this prospective observational study is to clarify whether an enhanced diagnostic MRI work-up combined with prolonged Holter-monitoring leads to a significant increase in relevant pathologic findings compared to guideline-recommended routine diagnostic work-up in patients with acute ischemic stroke without known atrial fibrillation before enrolment. There is a vital need to assess potential improvements in stroke care, because the etiology of about 20–25 % of all ischemic strokes remains unknown [4]. The high percentage of unknown stroke etiology may be explained by low rates of transesophageal echocardiography [1, 15] and insufficient duration of ECG monitoring [12]. Compared to transesophageal echocardiography, cardiac MRI and MR angiography of the aortic arc are less invasive and show growing potential for a prominent diagnostic role in acute ischemic stroke [6]. However, due to lack of prospective studies, there are no clear cut recommendations for their clinical use in this context so far. This study aims to evaluate the efficacy of MRI in detecting embolic sources of stroke and thereby elucidate its future relevance for clinical routine.

Besides feasibility and diagnostic value of enhanced MRI, HEBRAS will assess the ability of extended systematic analysis of ECG-recording to improve AF detection compared to the current clinical standard, as similarly done in currently ongoing trials such as the *Impact of Standardized MONitoring for Detection of Atrial Fibrillation in Ischemic Stroke* Study (MonDAFIS) [NCT02204267] or the Find-AF_RANDOMISED_ trial [27], as well as previous studies with shorter periods of monitoring [28, 29]. Detection of paroxysmal AF in patients with acute ischemic stroke represents a major diagnostic challenge nowadays, and AF remains undetected in a relevant proportion of stroke patients [9, 10]. A faster assessment and better understanding of stroke etiology may improve secondary stroke prevention measures and thereby help to reduce the rate of stroke recurrence.

Another unique feature of the investigator-initiated HEBRAS study is the systematic assessment of stroke-induced autonomic dysfunction and cardiac damage by using a laboratory data. It has long been recognized that acute cerebrovascular events may coincide with or trigger cardiac dysfunction and elevation of cardiac biomarkers such as troponin. Several studies reported on elevation of cTn in patients with acute ischemic stroke [30–32]. However, although cTn is highly specific for myocardial injury, it does not reveal the underlying mechanism of injury. Elevations in cTn do not necessarily signify coronary myocardial ischemia. There are potential other causes of cTn elevations without underlying acute or chronic CAD [33]. One possible mechanism of acute cardiac injury in ischemic stroke, which pertains exclusively to patients with acute disease of the central nervous system, is neurogenic myocardial damage. Neurogenic myocardial damage may be caused by impairments within the central autonomic network. A crucial player of the central autonomic network would be the insula. Within a previous work, we have shown a positive association between stroke affecting the insula and troponin elevation [31]. Therefore, HEBRAS will elucidate the relationship between stroke location and troponin elevation with measures of voxel based lesion mapping.

In addition, natriuretic peptides (e.g., BNP) have been shown to be independently associated with atrial fibrillation in stroke cohorts [34–36], but have not been evaluated in large prospective trials thus far.

## Conclusion

In summary, the HEBRAS study aims to clarify whether recent technical advantages can improve etiologic work up after acute ischemic stroke. This study has the potential to set essential modifications of current diagnostic standards after acute ischemic stroke, aiming at improving secondary stroke prevention. Moreover, the HEBRAS study will hopefully elucidate mechanisms of stroke-induced autonomic dysfunction and cardiac damage.

## Abbreviations

AF: Atrial fibrillation
bpm: Beats per minute
ECG: Electrocardiogramm
ESUS: Embolic Stroke of undetermined Source
FOV: Field of view
HEBRAS: HEart and BRain interfaces in Acute ischemic Stroke
HRV: Heart rate variability
HRV-TI: HRV-Triangular Index
LF: Low frequency
HF: High frequency
MRI: Magnetic resonance imaging
NASCET: North American symptomatic carotid endarterectomy trial
NYHA: New York heart association
s: Seconds
rMSSD: Root mean square of successive differences
SD-NN: Standard deviation of beat-to-beat intervals
TE: Echo time
TEE: Transesophageal echocardiography
TOAST: Trial of ORG 10172 in acute stroke treatment
TR: Repetition time
TTE: Transthoracal echocardiography
